# Fluorescent Receptor Binding Assay for Detecting Ciguatoxins in Fish

**DOI:** 10.1371/journal.pone.0153348

**Published:** 2016-04-13

**Authors:** D. Ransom Hardison, William C. Holland, Jennifer R. McCall, Andrea J. Bourdelais, Daniel G. Baden, H. Taiana Darius, Mireille Chinain, Patricia A. Tester, Damian Shea, Harold A. Flores Quintana, James A. Morris, R. Wayne Litaker

**Affiliations:** 1 National Oceanic and Atmospheric Administration, Center for Coastal Fisheries and Habitat Research, Beaufort, North Carolina, United States of America; 2 University of North Carolina at Wilmington, MARBIONC at CREST Research Park, Wilmington, North Carolina, United States of America; 3 SeaTox Research Inc, UNCW CREST Research Park, Wilmington, North Carolina, United States of America; 4 Institut Louis Malardé (ILM)–UMR 241 EIO, Laboratory of Toxic-Microalgae, Papeete, Tahiti, French Polynesia; 5 JHT, Inc., Orlando, Florida, United States of America; 6 North Carolina State University, Environmental Chemistry and Toxicology Laboratory, Raleigh, North Carolina, United States of America; 7 U.S. Food and Drug Administration, Division of Seafood Science and Technology, Gulf Coast Seafood Laboratory, Dauphin Island, Alabama, United States of America; Universidad de La Laguna, SPAIN

## Abstract

Ciguatera fish poisoning is an illness suffered by > 50,000 people yearly after consumption of fish containing ciguatoxins (CTXs). One of the current methodologies to detect ciguatoxins in fish is a radiolabeled receptor binding assay (RBA_(R)_). However, the license requirements and regulations pertaining to radioisotope utilization can limit the applicability of the RBA_(R)_ in certain labs. A fluorescence based receptor binding assay (RBA_(F)_) was developed to provide an alternative method of screening fish samples for CTXs in facilities not certified to use radioisotopes. The new assay is based on competition binding between CTXs and fluorescently labeled brevetoxin-2 (BODIPY^®^- PbTx-2) for voltage-gated sodium channel receptors at site 5 instead of a radiolabeled brevetoxin. Responses were linear in fish tissues spiked from 0.1 to 1.0 ppb with Pacific ciguatoxin-3C (P-CTX-3C) with a detection limit of 0.075 ppb. Carribean ciguatoxins were confirmed in Caribbean fish by LC-MS/MS analysis of the regional biomarker (C-CTX-1). Fish (N = 61) of six different species were screened using the RBA_(F)_. Results for corresponding samples analyzed using the neuroblastoma cell-based assay (CBA-N2a) correlated well (R^2^ = 0.71) with those of the RBA_(F)_, given the low levels of CTX present in positive fish. Data analyses also showed the resulting toxicity levels of P-CTX-3C equivalents determined by CBA-N2a were consistently lower than the RBA_(F)_ affinities expressed as % binding equivalents, indicating that a given amount of toxin bound to the site 5 receptors translates into corresponding lower cytotoxicity. Consequently, the RBA_(F)_, which takes approximately two hours to perform, provides a generous estimate relative to the widely used CBA-N2a which requires 2.5 days to complete. Other RBA_(F)_ advantages include the long-term (> 5 years) stability of the BODIPY^®^- PbTx-2 and having similar results as the commonly used RBA_(R)_. The RBA_(F)_ is cost-effective, allows high sample throughput, and is well-suited for routine CTX monitoring programs.

## Introduction

Ciguatera fish poisoning (CFP) results from consumption of fish that have accumulated lipid-soluble ciguatoxins (CTXs) produced by microalgae of the genus *Gambierdiscus*. With an estimated 50,000 cases a year worldwide, CFP is the most common non-bacterial seafood illness associated with eating fish [1]. CFP intoxication most often occurs in tropical and subtropical regions, but can occur anywhere given the wide distribution of fish harvested from the affected regions. CTXs bind voltage-gated sodium channels causing an influx of Na^+^ into the cell, disrupting cellular functions including signal transmission in nerves [2]. Typical CFP intoxication can cause gastrointestinal, cardiovascular (bradycardia with hypotension), and neurological (paresthesia) symptoms that can last from weeks to months, including the diagnostic hot cold temperature reversal (dysesthesia) [3].

Currently, there are no regulatory limits for CTX in fish but a guidance level of 0.01 ppb P-CTX-1 and 0.1 ppb C-CTX-1 equivalents for Pacific and Caribbean ciguatoxins, respectively, have been issued by the United States Food and Drug Administration (US FDA) [4, 5]. These recommendations were based on a 10-fold reduction of the lowest concentration of CTX in meal remnants found to cause human illness. Though many CTX congeners have been profiled, it has been difficult to develop globally accepted regulatory concentrations of CTXs and standardized methods for its analysis in seafood due to the lack of certified standards. Because of this limitation, most monitoring efforts use a two tier approach to monitor fish samples for ciguatoxins. The first step involves screening fish extracts using a functional assay, either a radiolabeled receptor binding (RBA_(R)_) or a neuroblastoma cell based assay (CBA-N2a). These assays are sensitive and require low concentrations of toxin standard [6, 7]. The second step involves confirming the presence of CTXs by liquid chromatography tandem mass spectrometry (LC-MS/MS) [7]. Each method has its own advantages and disadvantages.

The CBA-N2a is the most sensitive functional assay. This higher level of sensitivity is achieved by incubating a neuroblastoma cell line (Neuro-2a) with ouabain (O), which inhibits the Na^+^/K^+^-ATPase ion pump that transports Na^+^ out of the cell and veratridine (V), which holds sodium channels in a modified open position [8, 9]. Once pretreated in this manner, concentrations of CTX in the ppt range cause the death of neuroblastoma cells in a dose dependent manner. Because untreated Neuro-2a cells are natively resistant to even high levels of CTX, controls lacking O/V pretreatment are used to quantify cell mortality caused by non-sodium channel binding compounds present in the extracts. After the incubation of samples with toxin for 20–24 h, cell viability is determined by using a colorimetric assay. Fish samples are routinely screened for toxicity by this method [7]. The major disadvantages of this assay are the analysis time (2.5 days), the maintenance of the Neuro-2a cells, and the lack of a universally standardized protocol for conducting the assay [8].

The RBA_(R)_ represents an alternative functional method for estimating CTX concentrations and has been used successfully in recent years [6, 10]. It involves incubating isolated rat brain synaptosomes containing abundant Na^+^ channels with a fixed amount of tritiated PbTx-3 ([^3^H]-PbTx-3). Unlabeled PbTxs present in the samples compete quantitatively with the radiolabeled [^3^H]-PbTx-3 for site 5 on the sodium channel receptor [6, 11, 12]. Consequently, the binding of radiolabeled PbTx-3 is inversely related to the concentration of unlabeled PbTxs in the sample. Because CTXs and PbTxs bind to the same sodium channel receptor site, the PbTx assay could be adapted to measure CTXs in fish tissues [13]. A major advantage of the RBA_(R)_ is that it only requires three hours to complete after the tissue extract is prepared, compared to 2.5 days for the CBA-N2a [14]. The PbTx necessary to produce the radiolabeled conjugate ([^3^H]-PbTx-3), unlike CTXs, is also cost effective to produce. The RBA_(R)_ disadvantages are its lower sensitivity compared to the CBA-N2a, and the required radioactivity license and applicable regulations in the United States, European Union and certain other countries. RBA_(R)_ costs are significant in association with maintenance of an active radioactive license and mandatory safety precautions. Many laboratories in regions where these regulations apply lack the necessary license which limits its universal applicability.

However, a newly designed receptor binding assay was used with purified PbTx standards to demonstrate that fluorescently labeled PbTx-2 (BODIPY^®^- PbTx-2) exhibited the same binding kinetics to site 5 on sodium receptors as radioactively labeled ([^3^H]-PbTx-3) [15, 16]. Our data indicated the disadvantages of the RBA_(R)_ associated with using a radioactive ligand can be eliminated. Using a CTX standard, these studies also demonstrated the fluorescent receptor binding assay (RBA_(F)_) could be used to detect CTXs similar to the RBA_(R)_. The BODIPY^®^- PbTx-2 conjugate has the additional benefits of being highly stable (> 5 years) and a lower non-specific binding background which reduces variation between replicates when compared to the RBA_(R)_.

The goal of this study was to fully demonstrate the RBA_(F)_ as a rapid, reliable monitoring method for detecting CTXs in fish. This implementation required extensive method development to create a standardized extraction procedure and comparison of the assay results with those from RBA_(R)_ and CBA-N2a using extracts from representative samples of Caribbean fish. Positive CTX-like test results were then confirmed by mass spectrometry.

## Methods

### Reagents and Materials

All reagents used in this study were ACS grade or higher. Solvents were HPLC grade or higher purity. Caribbean ciguatoxin 1 (C-CTX-1) was purchased from Richard Lewis (University of Queensland, Australia). Pacific ciguatoxin 3C (P-CTX-3C) was purchased from WAKO Chemicals, USA, Inc. and also provided by Institut Louis Malardé (ILM). Brevetoxin 2 (PbTx-2) and BODIPY^®^-PbTx-2 were acquired from University of North Carolina at Wilmington (UNCW). All water used was Milli-Q Ultra-pure grade with 18.2 MΩ resistivity. For preparations of synaptosomes, frozen adult male Sprague-Dawley rat brains were purchased from Lampire Biological Laboratories, Pipersville, Pennsylvannia. Representative frozen whole fish or tissue samples were provided for analysis from fishery managers in charge of each study area.

### Fish Extraction for RBA_(F)_

Tissue samples were extracted using a modified protocol developed for the RBA_(R)_ [10]. Briefly, whole fish or fillets were thawed to room temperature and triplicate 5 g subsamples of flesh were removed from each specimen, placed in a 50 mL conical centrifuge tubes and cooked at 70°C for one hour. This step eliminated native fluorescence in the samples which can interfere with signal to noise ratios of the RBA_(F)_. Each tissue sample was then extracted with 7 mL of methanol by homogenizing with a finger sonicator (Q-Sonica, Q700, Newtown, Connecticut) for 1 minute. The tissue-containing tubes with methanol were capped and placed in a water bath sonicator at room temperature (Branson, 1800, Danbury, Connecticut) for two hours. After sonication was completed, the tissue-containing tubes were removed from the sonicator and held at room temperature overnight (14–16 h). The extracts were centrifuged at 4816 × g for 10 minutes. The supernatant from each tube was decanted into a 20 mL glass scintillation vial and adjusted to 70% methanol:30% water. Each supernatant was passed through a Waters Sep-Pak^®^ Plus C_18_ solid phase extraction (SPE) columns (WAT020515, 360 mg) preconditioned with 10 mL of 70% methanol:30% water using a Supelco visiprep™ (St. Louis, Missouri) DL vacuum manifold. After extracts were loaded onto SPE columns, they were washed with 70% methanol:30% water twice (7 mL × 2) and eluted with 7 mL of 90% methanol:10% water into a glass scintillation vial. The vials were transferred to a nitrogen evaporator (Organomation Associates, Inc., N-EVAP 111, Berlin, Massachusetts) and volumes were reduced to less than 2 mL under ultra-high purity nitrogen at 50°C. The concentrated extracts were transferred into 2 mL glass HPLC vials and blown to dryness, sealed and stored at -20°C until analysis. Just prior to running an assay, the dried extracts were resuspended as described below in sections *Fish RBA*_*(F)*_ *for Samples Containing > 1*.*0 ppb of CTX* or *Fish RBA*_*(F)*_ *for Samples Containing < 1*.*0 ppb of CTX*.

Selected fish tissues from each species of fish were spiked with P-CTX-3C and processed to determine extraction recovery efficiencies (83 ± 2%) and to confirm that the assay had a linear response over the 0.1–1.0 ppb P-CTX-3C equivalents range. Though having similar properties, P-CTX-3C may not extract with the same efficiency as C-CTX-1. Consequently, until sufficient quantities of C-CTX-1 become available, the precise extraction efficiency of this compound remains unknown and Pacific ciguatoxin standards will have to serve as a surrogate for extraction efficiency.

### Synaptosome Preparation

Synaptosomes rich in sodium channel receptors, which serve as the target for the assay, were prepared using a modification of the [12] procedure as described in [15]. Briefly, frozen adult male Sprague-Dawley rat brains were thawed on ice and homogenized in buffer (0.32 M sucrose, 0.005 M sodium phosphate, 0.02% protease inhibitor cocktail and brought to pH 7.4 with Trizma base) with a motor driven Teflon/glass homogenizer. The resulting homogenate was sedimented by centrifugation (700 × g for 10 min at 4°C) and the supernatant was saved. The supernatant was layered over a 1.2 M sucrose solution and centrifuged at 105,000 × g for 30 min at 4°C. The material at the interface was collected, layered over a 0.8 M sucrose solution and centrifuged at 140,000 × g for 35 min at 4°C. The supernatant was discarded and the final pellet containing synaptosomes was resuspended and diluted to 1 mg protein mL^-1^, and aliquots were stored at -80°C for use in subsequent assays. Protein content was assessed using a modified Lowry Protein Assay (Bio-Rad, Hercules, California). Synaptosomes can be used only once after transferring to a filter plate, and appear to retain optimal binding for up to 6 hours after thawing if kept cold. Therefore, synaptosomes were allocated into 1 mL cryovials in sufficient quantities to run one 96-well plate. Synaptosomes retain consistent binding for up to 6 months if kept at -80°C.

### RBA_(F)_ CTX Standard Curves for Estimating CTX in Fish Containing > 1.0 ppb P-CTX-3C Equivalents

In this study, RBA_(F)_ standard curves were constructed using purified P-CTX-3C ranging from 100–0.01 ng mL^-1^. Specifically, 100 ng of P-CTX-3C was dissolved in 10 μL of 200 proof ethanol to yield a stock concentration of 10 ng μL^-1^. The entire volume of this solution was used for the first standard to produce an in assay concentration of 100 ng mL^-1^. Another 100 ng of P-CTX-3C was dissolved into 100 μL of 200 proof ethanol to produce a stock concentration of 1 ng μL^-1^. This stock solution was the second standard (10 ng mL^-1^) and subsequent 1:10 dilutions beginning with this stock solution were completed in 200 proof ethanol to construct the remaining toxin standard curve. To test how the kinetics of the RBA_(F)_ compared with previously published RBA_(R)_, a limited number of standard curves were similarly produced using C-CTX-1 (RBA_(F)_) and P-CTX-3C (RBA_(R)_) from stock solutions of 1 ng μL^-1^ over a concentration range of 10–0.01 ng mL^-1^. The 100 ng mL^-1^ standard was omitted for these two curves due to limited quantities of standards.

### RBA_(F)_ Standard Curves for Estimating CTX in Fish Containing < 1.0 ppb P-CTX-3C Equivalents

Most of the fish surveyed by RBA_(F)_ in this study contained < 0.2 ppb P-CTX-3C equivalents, a concentration too low for an accurate IC_50_ estimate due to an incomplete sample dilution curve that does not reach 50% binding. When 50% binding is not achieved on dose response curves, the analysis software inaccurately estimates the IC_50_ despite its extrapolation feature. We discovered that CTX concentrations in these low toxicity fish could alternatively be precisely assessed by generating a linear standard curve. Previously tested nontoxic 5 g fish extracts of each fish species were spiked with sufficient P-CTX-3C to yield concentrations equivalent to 0.1, 0.25, 0.5, and 1.0 ng toxin g^-1^ fish extract. Additions to the assay were at the 2 g mL^-1^ fish tissue concentration for each of these spiked extracts to produce the linear curve. The correlation of the % binding versus corresponding P-CTX-3C concentration was linear with an R^2^ > 0.99 and highly reproducible among experiments. For routine screening of Caribbean samples, the RBA_(F)_ was conducted using these standard curves and duplicate aliquots from each sample at the 2 g mL^-1^ fish extract concentration also. This approach allowed a large number of samples to be screened simultaneously, and all but one fish examined in this study could be quantified using this method. This particular fish was high enough in CTX concentration that a full dilution curve was possible, and analysis was done as described later in section *Fish RBA*_*(F)*_ *for Samples Containing > 1*.*0 ppb of CTX*.

### RBA_(F)_ Protocol

The RBA_(F)_ developed in this study was based on the protocol of [15]. Gilson MICROMAN^®^ positive displacement pipettes (Gilson, Inc., Middleton, Wisconsin) were used to transfer all the organic solutions used in the assay for greater accuracy. The assay buffer contained 50 mM HEPES, 130 mM choline chloride, 5.4 mM KCl, 1.7 mM MgSO_4_, 5.5 mM glucose, 6.1 mM ethylene glycol, bovine serum albumin 1 g L^-1^, 3 to 4 g L^-1^ Trizma^®^ base was added to achieve pH 7.4, and protease inhibitor cocktail (P-8340) 200 μL L^-1^ (All reagents from Sigma Aldrich, St. Louis, Missouri). The buffer was stored at 4°C. For each plate, 100 mL of assay buffer was combined with one drop of Tween-20 detergent (~ 0.02%,) in a glass bottle and mixed using a stir bar at room temperature for 15–20 min. The BODIPY^®^- PbTx-2, which competes with CTXs in the sample for the synaptosome sodium channel sites, was dissolved in 200 proof ethanol to produce a 0.1 mM solution and stored at -20°C in the dark. Prior to running an assay, 2 μL of the BODIPY^®^- PbTx-2 solution was added per 10 mL of assay buffer in low light and the mixture vortexed for one minute.

Once the CTX standards, assay buffer, BODIPY^®^- PbTx-2, and sample extracts were prepared, the assay was assembled in a deep well polystyrene 96-well assay plate (PALL AcroPrep™, Ann Arbor, Michigan) as follows. The outer wells of the 96-well plate were not used due to high variability. First, 200 μL of either diluted toxin standard or sample extract were added to duplicate wells. Next, 50 μL each of synaptosomes and BODIPY^®^- PbTx-2 solution were added to each well using a multichannel pipette. Finally, 200 μL of buffer was added for a total assay volume of 500 μL per well. The combined reagents were sealed with 96-well plate film and were allowed to incubate on ice in the dark (Styrofoam cooler) while gently mixing (80 rpms) on a shaker (Thermo Scientific, Max Q 2000) for 2 hours. This allowed the CTX present in the standards or samples to compete with the BODIPY^®^- PbTx-2. At the end of the incubation, Pall AcroPrep^TM^ Advance 350 1 μm glass fiber filter plates (96-well format) were placed on a Pall^®^ multi-well plate manifold vacuum apparatus. A 250 μL aliquot of assay buffer mix was added to each well in the plate and then aspirated through the filter under vacuum (10 cm Hg). Under these conditions, it took 10–15 seconds for the assay buffer to pass through the membrane at the bottom of each well. To ensure that the plate was completely dry, the vacuum was released; the plate was removed from the vacuum manifold and tapped 3–4 times to remove any residual fluid retained on the side of the wells. The plate was then placed back on the manifold and a vacuum applied for another 10–15 seconds, the vacuum was released and the plate was blotted on the paper towel. This process was then repeated a third time.

Next, reaction mixtures prepared in the polystyrene 96-well plates were transferred to the buffer-washed filter plate mounted in the vacuum manifold, and filtered using the exact methods list above and read using a FLUOstar Omega fluorometer (BMG Labtech, Germany) with a 505 nm long band pass dichroic filter, a 490–10 nm excitation filter and a 520–10 nm emission filter to obtain the relative fluorescence units (RFUs) for each well. Percent binding of BODIPY^®^- PbTx-2, expressed as RFUs, decreased with increasing toxin concentrations. The lowest RFU value for the highest P-CTX-3C standard was subtracted from each well to account for any background fluorescence due to non-specific binding of the BODIPY^®^- PbTx-2 to the synaptosomes or filter plate. The data were then scaled so that the corrected RFUs from the standard curve and samples containing no P-CTX-3C represented 100% binding and the RFUs from the highest P-CTX-3C concentration was set to 0% binding. The normalized RFUs versus concentration data were fitted to a 4 parameter logistic model (= 4PL, Hill Slope Model) to estimate IC_50_ values using Graphpad software (GraphPad Prism version 6 for Windows, GraphPad Software, San Diego California, www.graphpad.com). The time required for the RBA_(F)_ to reach equilibrium was tested by letting the assay incubate for different periods of time ranging from 1.5 to 4 hours.

### Fish RBA_(F)_ for Samples Containing > 1.0 ppb of CTX

The dried extracts from 5 g of fish tissue were resuspended in 1000 μL of 10% methanol: 90% water and a 6-point dilution series of this solution was carried out as described above in sections *RBA(F) CTX Standard Curves for Estimating CTX in Fish Containing > 1*.*0 ppb P-CTX-3C Equivalents* and *RBA*_*(F)*_ *Protocol*. The only exception was that the fish extract dilution series utilized a 1:3 dose response curve beginning at 2 g mL^-1^ fish extract concentration rather than the 1:10 used for the standards. The resultant % binding and corresponding log fish extract concentration (g mL^-1^) were entered into GraphPad Prism to determine an IC_50_ value (g fish extract mL^-1^). Then the P-CTX-3C equivalents (ng per g fish tissue; ppb) present in the fish were calculated by dividing the P-CTX-3C IC_50_ (ng mL^-1^) by the extract IC_50_ (g fish tissue mL^-1^) [10].

### Fish RBA_(F)_ for Samples Containing < 1.0 ppb of CTX

The dried extracts from 5 g of fish tissue were resuspended in 1000 μL of 10% methanol: 90% water and the RBA_(F)_ was carried out in duplicate using a single point fish tissue concentration at 2 g mL^-1^. The resulting % binding was used to calculate the corresponding toxin concentration from the linear regression equation of the < 1.0 ppb P-CTX-3C standard curve described above (sections *RBA*_*(F)*_ *Standard Curves for Estimating CTX in Fish Containing < 1*.*0 ppb P-CTX-3C Equivalents* and *RBA*_*(F)*_ *Protocol*). Using the previously determined relationship between the P-CTX-3C and C-CTX-1 IC_50_ values, it was possible to convert the P-CTX-3C results to C-CTX-1 equivalents. The limit of detection and the limit of quantitation were experimentally established by spiking blank fish tissue samples with 0, 0.05, 0.075, and 0.1 ppb P-CTX-3C and determining at what tissue concentration yielded consistent and accurate % binding estimates.

### Radioactive Receptor Binding Assay (RBA_(R)_)

To determine if the relative binding kinetics of the RBA_(F)_ and RBA_(R)_ were equivalent, the RBA_(R)_ P-CTX-3C standard curves were conducted exactly like the RBA_(F)_ except that 2 nM of tritiated brevetoxin ([^3^H]-PbTx-3) was used as the site 5 competitor instead of BODIPY^®^- PbTx-2 [6, 11, 12]. After the filtration of the reaction mixtures, 35 μL of scintillation cocktail (Packard Microscint^TM^ 20) was added to each well and samples were counted on a Packard TopCount NXT liquid scintillation spectrometer. The resulting data was analyzed using GraphPad Prism as described in section *RBA*_*(F)*_ *Protocol* above.

### Fish Extraction for CBA-N2a

Another aspect of this study was the comparison of results of duplicate extracts from a variety of fish analyzed by both the RBA_(F)_ and CBA-N2a. Two separate extraction protocols were required to perform CBA-N2a depending on the type of fish analyzed. The previous extraction procedure for RBA_(R)_ analysis [10] was found to be sufficient for extraction of lionfish. The remaining species of fish required a more thorough extraction procedure to avoid nonspecific interference with the CBA-N2a. This second extraction protocol for CBA-N2a involved placing 5 g of fish tissue in conical 50 mL tubes and heating them at 70°C for one hour. Samples were then removed from the oven and extracted according to the protocol of [17]. Briefly, the 5 g of cooked fish tissue was extracted in 10 mL of methanol twice and then centrifuged at 4816 × g for 10 minutes to remove the toxin containing supernatant. The supernatants were then dried under nitrogen at 50°C, redissolved in dichloromethane and partitioned with 60% aqueous methanol. The dichloromethane fraction was collected in a 20 mL glass scintillation vial and dried under nitrogen. Next the dried samples were resuspended in 80% aqueous methanol and cyclohexane to remove lipids. Samples were allowed to separate overnight; the 80% aqueous methanol was collected, and dried under nitrogen. The dried samples were stored at -20°C pending analysis. Samples were redissolved in 200 μL methanol before using the CBA-N2a protocol.

### CBA-N2a Procedure

The Neuro-2a is frequently used to assess for CTX activity in fish or phytoplankton extracts [6, 8, 17]. This particular cell line was obtained from the American Type Culture Collection (ATCC CCL 131). Cells were grown and maintained in Eagle’s Minimum Essential Medium (EMEM; ATCC^®^ 30–2003) containing 2 mM L-glutamine, 1 mM sodium pyruvate, 100 μg mL^-1^ streptomycin, 100 units mL^-1^ penicillin, and 10% fetal bovine serum. Neuro-2a cells were kept at 37°C in a humidified 5% CO_2_ atmosphere. To prepare for toxicity analysis, Neuro-2a cells were harvested with a trypsin-(ethylenediaminetetraacetic acid) (EDTA) solution and seeded into each well of a 96-well microtiter plate at 30,000 cells per 100 μL of growth medium under the same growth conditions as above.

Plates seeded with Neuro-2a cells were allowed to settle and grow (20 to 24 h) until they were > 90% confluent at the bottom of each well. The standards, controls and samples were added next and incubated for 24 h. The P-CTX-3C standard curve for this assay ranged from 0.001–2,000 pg mL^-1^. The standard curve utilized 250 μM ouabain (O) and 25 μM veratridine (V) at 50% cell viability to increase sensitivity and specificity to CTX. Controls included buffer wells to provide maximum survival estimates and wells with the addition of 1% methanol (final concentration) to identify any cell mortality caused by the presence of methanol used to dissolve the dried extracts. Half of the sample aliquots (1 μL additions) from each assay were processed in the presence of ouabain and veratridine (O/V^+^) so they were directly comparable to the standard curve. The other half was incubated without ouabain and veratridine (O/V^-^) to identify non-specific mortality caused by other compounds in the sample. Total well volume was 100 μL. Cell viability was assessed after 20–24 h of toxin exposure at 37°C using the colorimetric 3-(4,5-dimethylthiazol-2-yl)-2,5-diphenyl tetrazolium bromide (MTT) assay [17].

### Spiked P-CTX-3C Fish Recoveries

To quantify toxin recovery during extraction for each species of fish, five grams of fish tissue was spiked with P-CTX-3C toxin to a concentration of 0.1 ng g^-1^ for both extraction processes ([10] - 83%; [17] - 90%). The tissue extractions were then conducted according to the protocol of [10] for the RBA_(F)_ or [17] for the CBA-N2a.

### Sampling Representative Fish Species

To test the versatility of the RBA_(F)_ in screening for ciguatoxins, we surveyed a total of 61 representative carnivorous fish samples whose habitats spanned the Caribbean including the following species: lionfish (*Pterois miles/volitans*, n = 37), horse eye jack (*Caranx latus*, n = 1), dog snapper (*Lutjanus jocu*, n = 8), schoolmaster snapper (*Lutjanus apodus*, n = 12), yellow goatfish (*Mulloidichthys martinicus*, n = 1) and yellow jack (*Carangoides bartholomaei*, n = 2). Resulting positive samples by RBA_(F)_ were analyzed further using CBA-N2a and LC-MS/MS.

### LC-MS/MS

Confirming the presence of C-CTXs in fish is based upon detecting its regional biomarker, C-CTX-1, by LC-MS/MS analysis. The analyses in fish samples with a composite toxicity > 0.1 ppb P-CTX-3C equivalents, determined by RBA_(F)_ and CBA-N2a were sent to the Food and Drug Administration (FDA), Gulf Coast Seafood Laboratory, Dauphin Island, Alabama. The confirmation of C-CTX-1 in the fish extracts was achieved by selecting the dehydrated C-CTX-1 ion (M + H–H_2_O)^+^ as a precursor for the following MRM ion transitions: *m/z* 1123.6 > 1105.6, 1123.6 > 1087.6, and 1123.6 > 1069.6. Quantitative C-CTX-1 measurements were not possible due to the limited quantity of FDA C-CTX-1 standard available, and the FDA’s reservation for its use in outbreaks of CFP when they occur. Furthermore, the relationship of C-CTX-1 to composite toxicity of CTXs as assessed by functional assays in various fish species has not been established.

#### Toxin Extraction for LC-MS/MS Analysis

Sample extraction was carried out according to a previous FDA method [7] with minor modifications. Muscle tissue sub-samples of 25 g were taken from a couple of the highest toxicity fish and extracted with acetone (2 mL g^-1^ tissue) using a blender (Eberbach, E8017, MI). Sample extracts were filtered through Whatman #4 filter paper. The acetone extraction was repeated on fish residue and filtrates were pooled and chilled at -20°C (at least 12 h). Extracts were then filtered through Whatman #5 filter paper and the residue discarded. The acetone extract was dried using rotary evaporation (Büchi R-210, Flawil, Switzerland) at 45°C. Dried residue from each extract was re-dissolved in 80% aqueous methanol (1 mL g^-1^ tissue) and partitioned three times with *n*-hexanes (1 mL g^-1^ tissue). Upper hexane layers were discarded and the aqueous methanol layer was collected, and dried by rotary evaporation at 45°C. The resulting residue was re-dissolved in water (3 mL g^-1^ tissue) and partitioned three times with chloroform (1 mL g^-1^ tissue). The upper water phase was discarded and the chloroform layers were collected, pooled and concentrated by rotary evaporation at 30°C before transferring to a scintillation vial and dried under nitrogen.

#### Solid Phase Extractions (SPE)

The SPE clean-up protocol used for sample extracts was based on [18]. Briefly, a 1 g amino cartridge (Bond Elut, Agilent, California) was conditioned with 6 mL of chloroform. Sample residues were re-dissolved in chloroform and loaded onto SPE cartridges. The cartridges were then washed with an additional 6 mL of chloroform, and eluted with chloroform:isopropanol (2:1). Eluants were collected and dried at 65°C. Final residues were redissolved in 500 μL methanol before LC-MS/MS analysis.

#### Qualitative LC-MS/MS Analysis of C-CTX-1

Confirmation of C-CTX-1 in this study was carried out using a modified protocol [18]. The analysis was performed on an Agilent 1260 LC system (Agilent Inc., Palo Alto, California) coupled to a QTRAP 4000 mass spectrometer (Applied Biosystems, Inc., Foster City, California). The analyte was eluted on a Kinetex C8 (75 × 2.1 mm; 2.6 μm) column (Phenomenex, Torrance, California) using a mobile phase consisting of A (water) and B (95% aqueous acetonitrile), both containing 0.1% formic acid. The mobile phase program consisted of 10% B for 1 min, then to 95% B at 1.5 min, held at 95% B for 5 min, and returned to 10% B in 0.2 min. The column was re-equilibrated with 10% B for 2.8 min. The flow rate was set to 0.3 mL min^-1^ and the column temperature and auto-injector tray were maintained at 40°C and 10°C, respectively. The injection volume was set at 5 μL. The mass spectrometer was operated in positive electrospray ionization mode. Principal instrument settings maintained were: CUR 20 psi, IS 5500 V, TEM 400°C, GS1and GS2 both at 60 psi; CAD medium; DP: 75 V, EP 10 V, CE 35 eV and CXP 15 V. Analyst 1.6.1 was used for data acquisition. C-CTX-1 standard used in the LC-MS/MS analysis was provided by the FDA.

## Results

### RBA_(F)_

**Fig 1:**
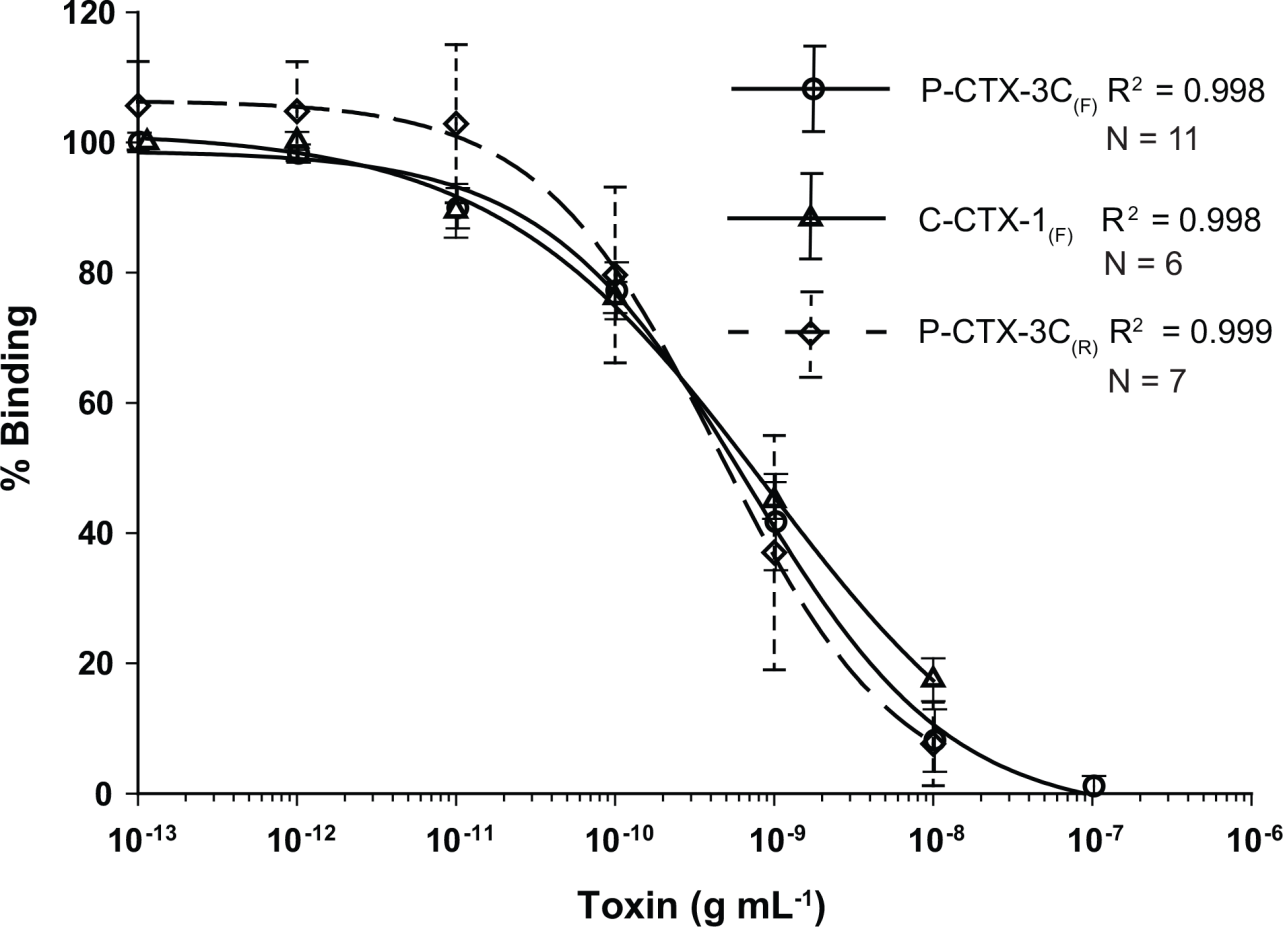
RBA_(F)_ and RBA_(R)_ Ciguatoxin Standard Binding Curves. Comparison showing the similarity of the binding kinetics (percent [%] binding vs. toxin concentration [g mL^-1^]) between the fluorescent (RBA_(F)_, open circle; solid line) and radioactive receptor binding (RBA_(R)_, open triangle; solid line) assays when P-CTX-3C was used as the standard. Also shown are the RBA_(F)_ binding kinetics when C-CTX-1 was used as the standard (open triangle; solid line). Error bars equal ± one standard deviation with solid lines representing RBA_(F)_ standard deviations and dashed lines for the RBA_(R)_ results.

The competitive binding of P-CTX-3C and C-CTX-1 to sodium channels found in isolated rat brain synaptosomes was measured quantitatively using both RBA_(F)_ and RBA_(R)_. Serial dilutions of these toxins resulted in sigmoidal dose response curves (Fig 1; all R^2^ > 0.998). The resulting IC_50_ estimate for P-CTX-3C RBA_(F)_ (0.66 ± 0.16 ng mL^-1^) was nearly identical to values previously reported for the RBA_(R)_ (Table 1) in [10] and [17]. In contrast, the average RBA_(R)_ estimate obtained in this study (0.35 ± 0.17 ng mL^-1^) was lower than prior studies (Table 1). This may have been due to the age of the radioligand, which resulted in higher background and increased replicate variability (dashed line error bars in Fig 1). Only one IC_50_ estimate is available for a C-CTX-1 RBA_(R)_ [19] and it is lower (0.34 ± 0.11 ng mL^-1^) than the RBA_(F)_ estimate from this study (0.87 ± 0.12 ng mL^-1^) (Table 1). The RBA_(F)_ ratio of the average IC_50_’s for C-CTX-1 to that of P-CTX-3C standards was 1.3 (Table 1; Fig 1) and can tentatively be used to convert P-CTX-3C to C-CTX-1 equivalents for Caribbean studies. This conversion is only an estimate as the 1.3 ratio relates to standards of one toxin congener, and samples likely have multiple congeners expressing activity.

**Table 1:** RBA_(F)_ and RBA_(R)_ IC_50_ Values. Literature and this study’s IC_50_ values of ciguatoxins P-CTX-3C and C-CTX-1 for fluorescent and radiolabeled receptor binding assays (RBA_(F)_ and RBA_(R)_). Error is represented as ± standard deviations (SD).

	IC_50_ (ng mL^-1^) ± SD	
RBA_(F)_	P-CTX-3C	C-CTX-1
This study	0.66 ± 0.16	0.87 ± 0.12
**RBA**_**(R)**_		
This study	0.35 ± 0.17	
[19]		0.34 ± 0.11
[10]	0.62 ± 0.16	
[17]	0.61 ± 0.01	

**Fig 2:**
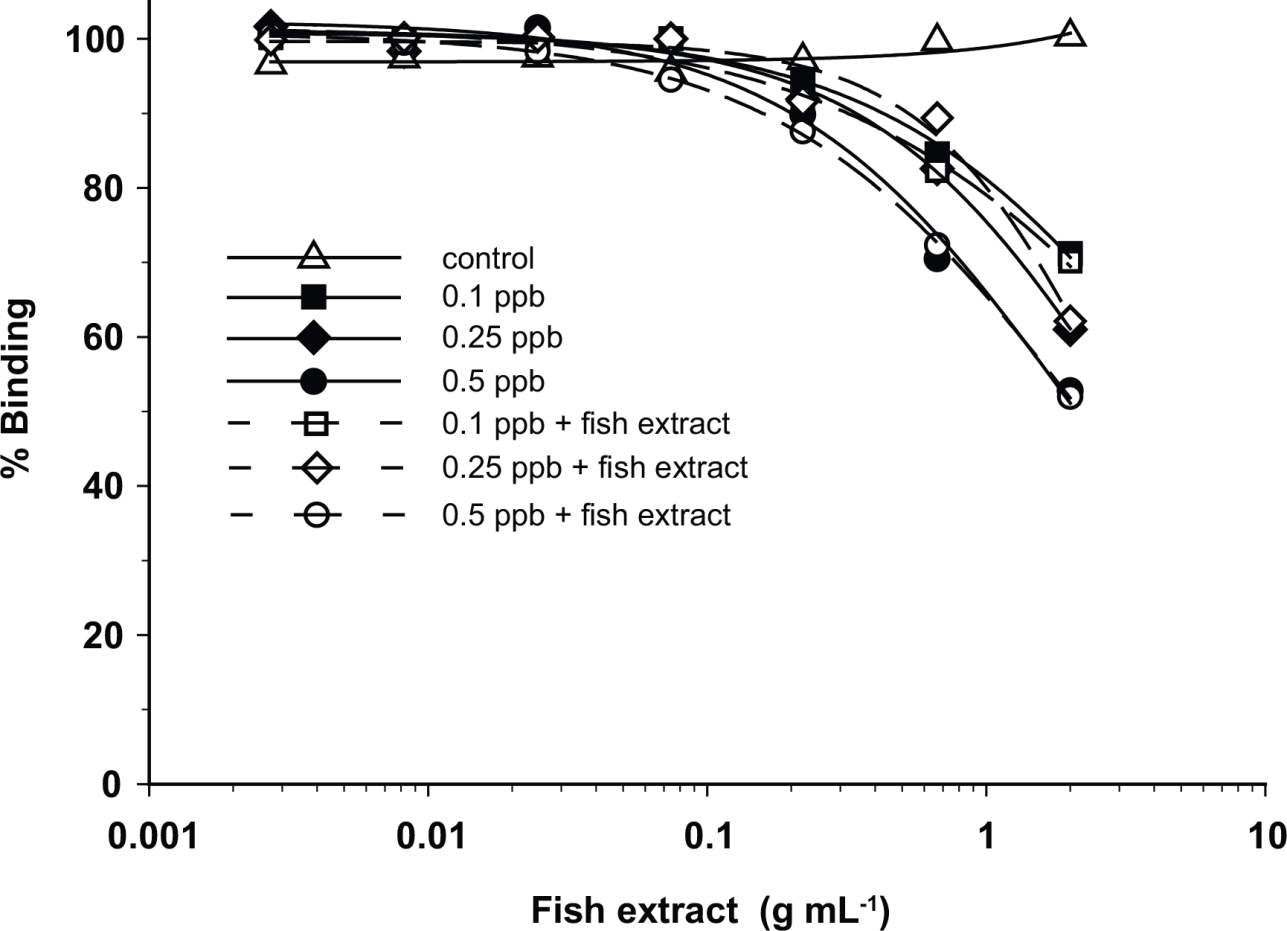
Solid-Phase Extraction Results. Separate 5 g fish extracts from a fish containing no measurable ciguatoxin were spiked to a final concentration of either 0.1 (open squares), 0.25 (open diamonds), or 0.5 (open circles) ppb P-CTX-3C. Comparable buffer samples were similarly spiked with of either 0.10 (solid squares), 0.25 (solid diamonds), or 0.50 (solid circles) ppb P-CTX-3C. The control was tissue extract with no ciguatoxin added. Spiked and control samples were serially diluted 1:3 and results plotted as % binding versus equivalents of fish tissue extracted (g mL^-1^). Results indicated that matrix effects in the fish extracts did not interfere with binding of P-CTX-3C.

**Fig 3:**
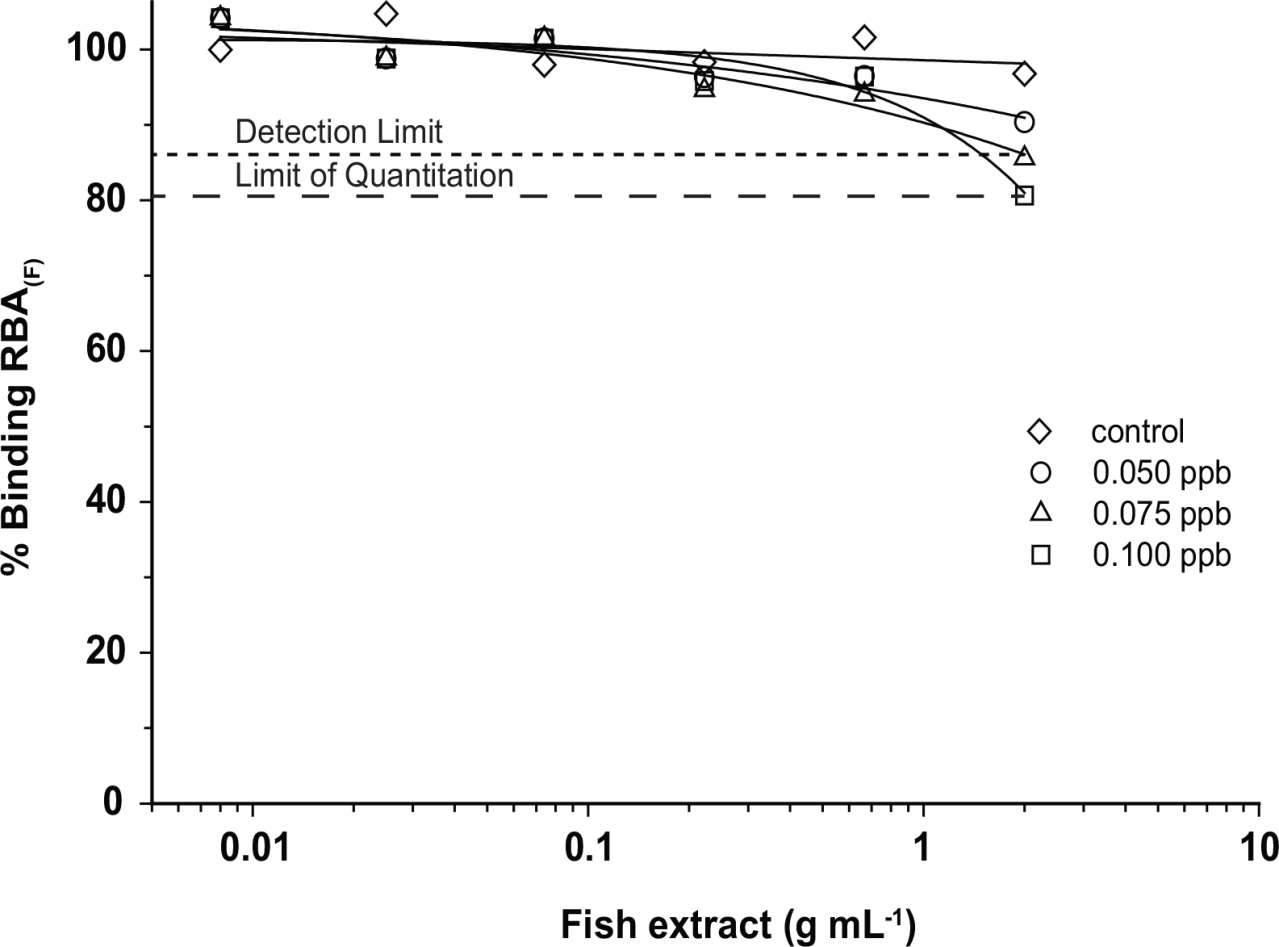
RBA_(F)_ Sample Binding Curves. Representative RBA_(F)_ curves of 1:3 serially diluted of fish samples spiked with 0 (open diamond), 0.050 (open circle), 0.075 (open triangle), and 0.100 ppb (open square) P-CTX-3C with percent (%) binding versus fish extract concentration (g mL^-1^). The detection limit (small dashed line) was 0.075 ppb P-CTX-3C and the limit of quantitation (larger dashed line) was 0.100 ppb P-CTX-3C.

**Fig 4:**
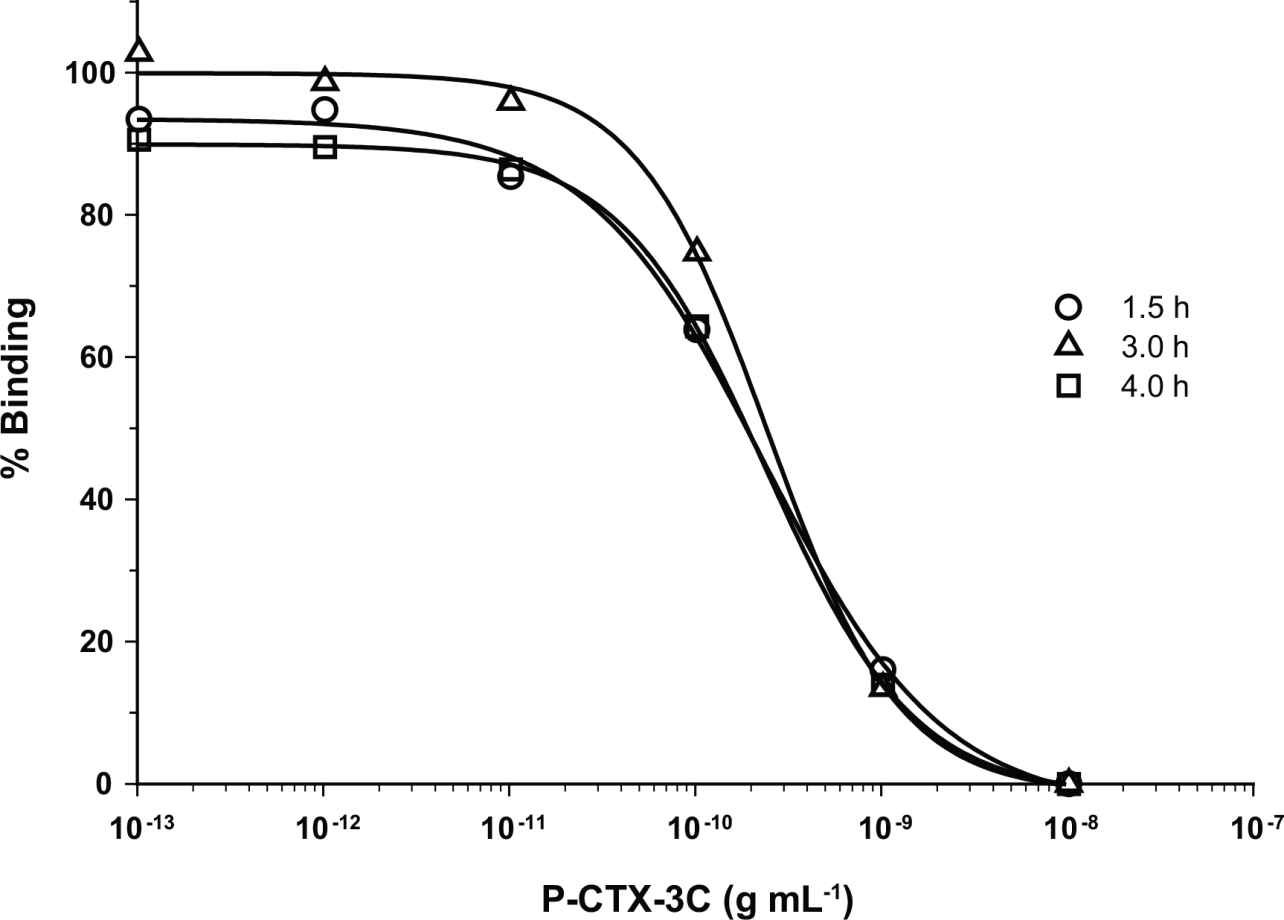
Time Series of RBA_(F)_ Reaction. RBA_(F)_ standard curves of percent (%) binding versus P-CTX-3C (g mL^-1^) where the standards were allowed to incubate with the synaptosomes for 1.5 (open circle), 3.0 (open triangle) and 4.0 h (open square).

Initially, RBA_(F)_ analysis of crude fish extracts revealed signal suppression of the analyte signal by matrix effects. This was manifested as an increase in fluorescence signal intensity above 100% binding with increasing concentration of fish extract in the assay. Nearly all of the background fluorescence was eliminated by heating the flesh at 70°C for one hour. The cooking process does not affect ciguatoxins concentrations as they have been found to be stable in cooked samples [20]. Spiking samples with P-CTX-3C further showed that crude extracts from the cooked fish contained additional compounds that quenched the fluorescence signal from the BODIPY^®^- PbTx-2. This interference was eliminated by using a C_18_ solid phase extraction (SPE) protocol developed for the RBA_(R)_ by [10]. Further spiked experiments showed (a) that samples cleaned up using the SPE column did not show significant matrix effects (Fig 2), (b) the experimental limit of detection was 0.075 ppb P-CTX-3C equivalents and (c) the experimental limit of quantitation was 0.1 ppb C-CTX-1 equivalents (Fig 3). Incubating the samples with BODIPY^®^- PbTx-2 for varying lengths of time also showed that binding was complete after 1.5 h (Fig 4). Given this information, the described SPE extraction, cooking procedures, and 1.5 h incubation time were adopted as the standard protocol.

**Fig 5:**
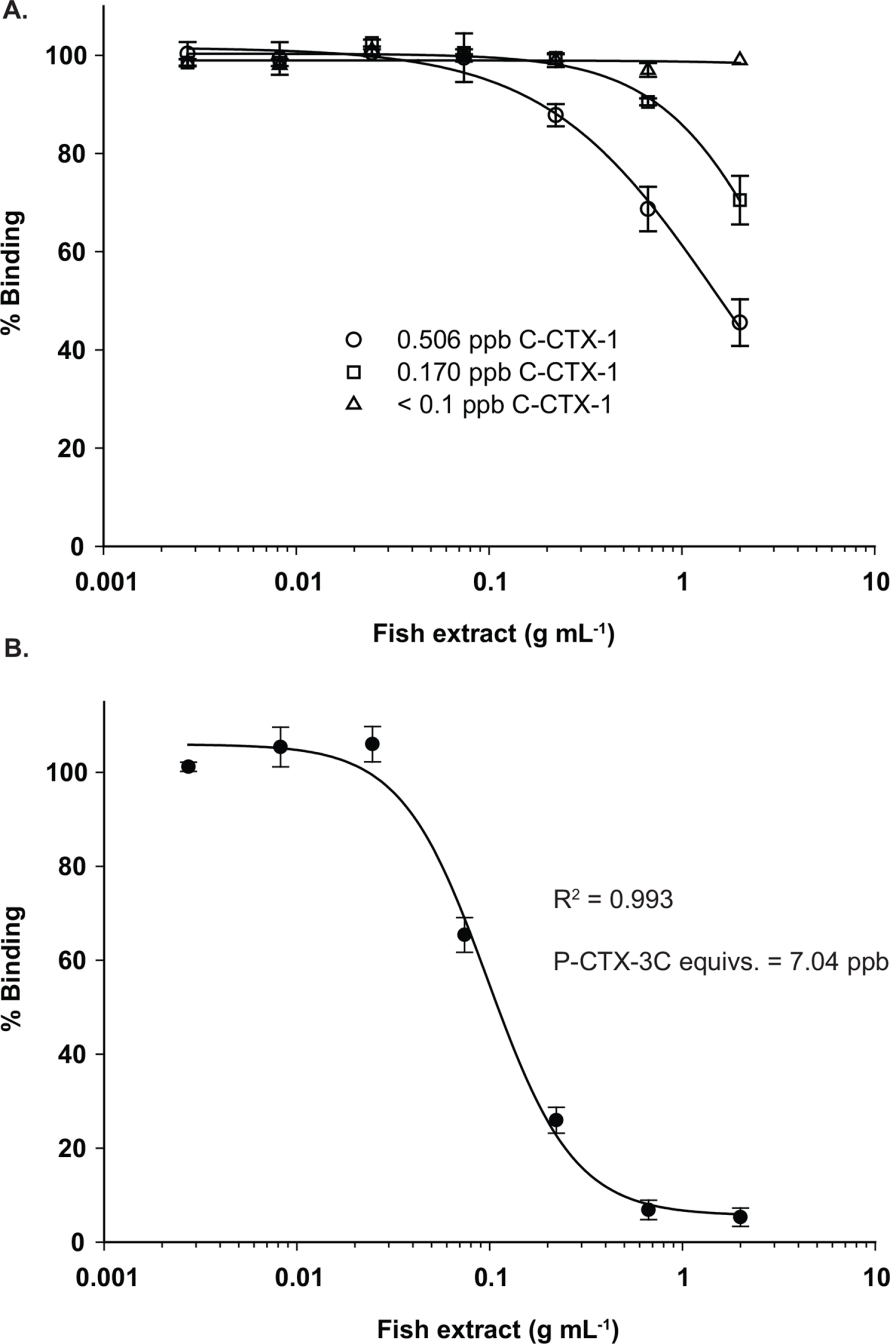
RBA_(F)_ Fish Sample Curves. (A) RBA_(F)_ 1:3 dilution series (% binding versus fish extract [g mL^-1^]) for different fish with 0.506 (open circle), 0.170 (open square), and < 0.100ppb (open triangle) C-CTX-1equivalents. Dilution curves of fish containing less than 0.500 ppb CTX do not reach 50% binding preventing accurate determination of the IC_50_ values. (B) RBA_(F)_ 1:3 dilution curve of a fish containing >7.00 ppb P-CTX-3C equivalents illustrating that the IC_50_ values from extracts containing higher CTX concentrations can be estimated accurately. Ciguatoxin concentrations in the fish are estimated by dividing the IC_50_ for the ciguatoxin standard curve (g ciguatoxin mL^-1^) by the IC_50_ from the diluted fish extract (g fish extracted mL^-1^).

**Fig 6:**
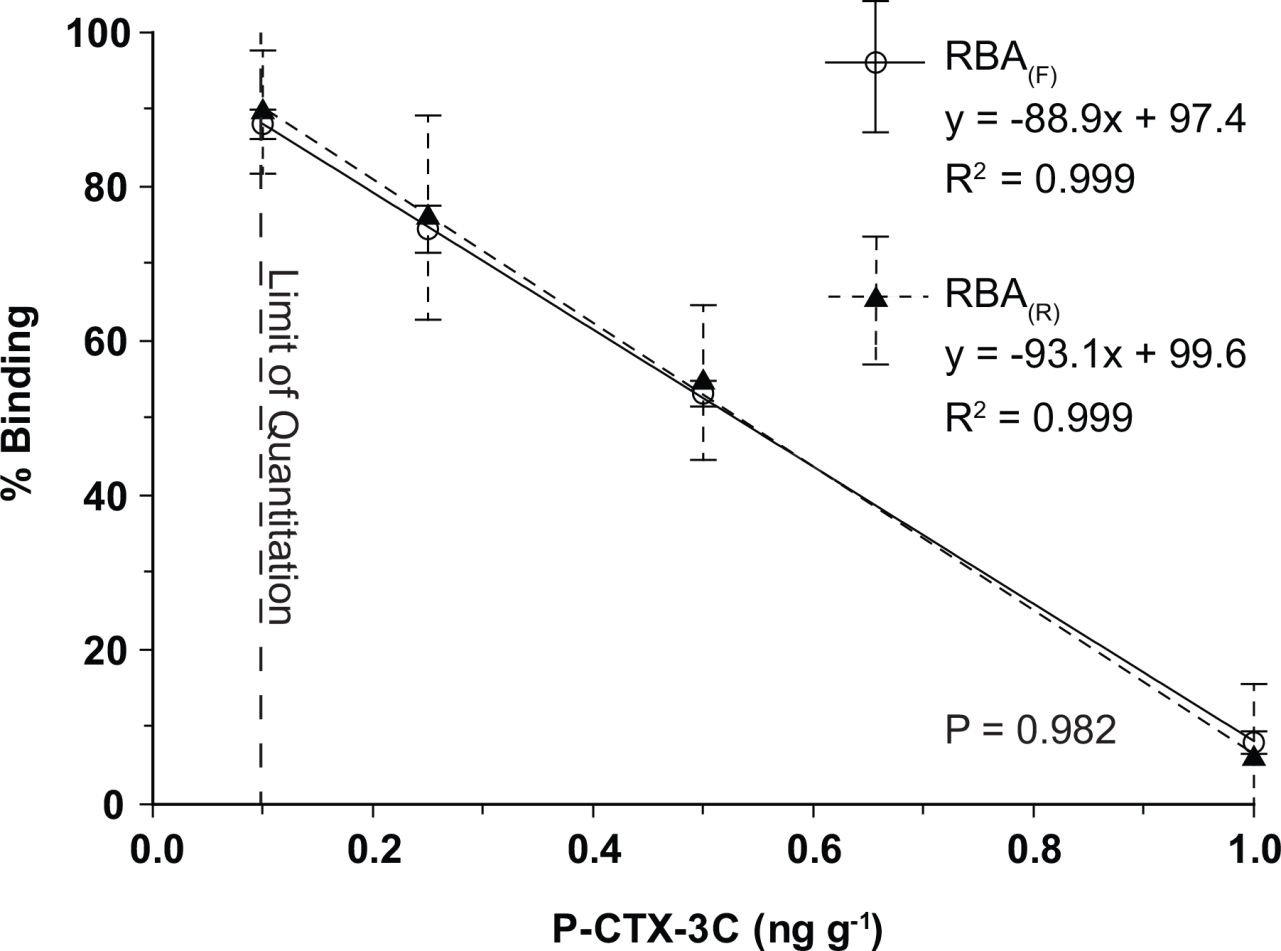
RBA_(F)_ and RBA_(R)_ Linear Standard Curves. Lower ciguatoxin concentrations found in fish surveyed in this study were accurately measured using a linear standard curve consisting of 0.10, 0.25, 0.50 and 1.0 ppb P-CTX-3C. Results were equivalent for the RBA_(F)_ (open circle; solid line) and RBA_(R)_ (closed triangle; dashed line) (t-test, P = 0.982).

Using both purified P-CTX-3C toxin standards and fish containing different concentrations of CTXs, it was possible to show that the vast majority of fish containing < 1.0 ppb P-CTX-3C equivalents should be quantified using the 0 to 1.0 ppb linear standard curve (Figs 5 and 6). The curve was linear over the concentration range of 0.1–1.0 ng g^-1^ and had a regression coefficient of 0.999 (Fig 6). The slope was not significantly different than the RBA_(R)_ curves obtained using the same samples (t-test, P = 0.982). Repeated experiments of CTX spiked fish samples, previously found to be nontoxic, further showed that reliable quantitative estimates between 0.1 and 1.0 ppb P-CTX-3C (= 0.13–1.3 C-CTX-1 equivalents) were possible. Semi-quantitative estimates can be achieved at 0.075 ppb P-CTX-3C equivalent (0.0975 ppb C-CTX-1), slightly below the level of 0.1 ppb C-CTX-1 equivalents [5]. Another advantage of using the 0–1.0 ppb P-CTX-3C curve is that accurate CTX concentrations can be determined by screening replicate sample aliquots from the most concentrated fish extracts (2 g mL^-1^) without constructing a full dilution curve thus making more plate wells available for samples. In instances where fish contained higher concentrations of CTX (> 1 ppb), a full sample dilution curve is needed to determine the IC_50_. Final fish CTX concentration is then determined by dividing the IC_50_ (ng of CTX mL^-1^) of the CTX standard with that of the sample IC_50_ (g fish mL^-1^) to yield ng of CTX g^-1^ sample (Fig 5B).

### CBA-N2a

**Fig 7:**
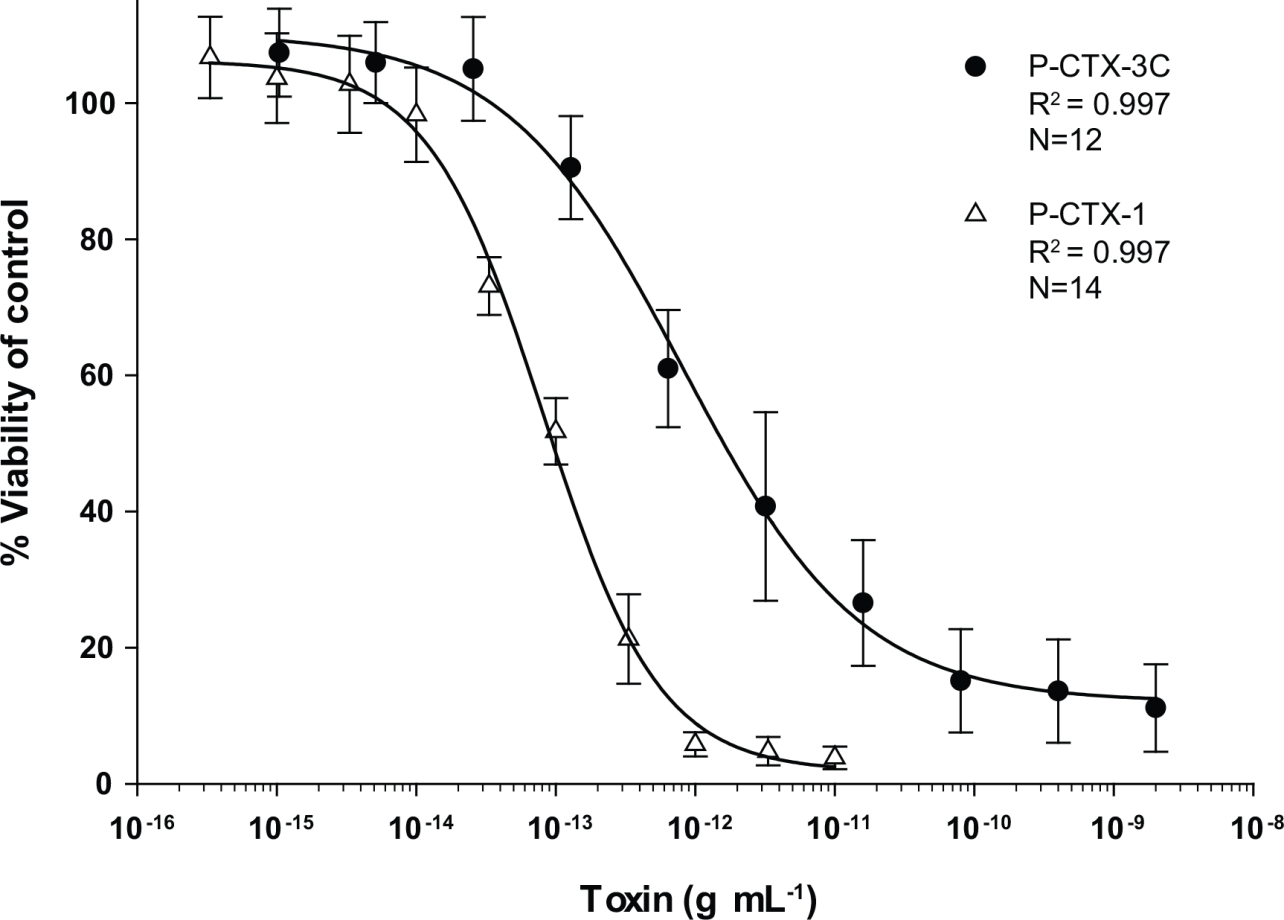
CBA-N2a Ciguatoxin Standard Curves. CBA-N2a standard curves for P-CTX-3C (closed circle) and P-CTX-1 (open triangle) with percent (%) viability of Neuro-2a plotted against toxin concentration (g mL^-1^). Percent cell viability is defined as the survival ratio of cells treated with and without ouabain and veratridine. Error bars represent ± standard deviations of multiple standard curves. The number of curves included is given as (N).

The CBA-N2a was also used as a secondary test of the RBA_(F)_ to validate results. CBA-N2a standard curves for P-CTX-1 and P-CTX-3C are shown in Fig 7. As toxin concentration increases, cell viability of O/V^+^ treated cells versus O/V^-^ control cells decreases in a dose dependent relationship. Both standard curves had regression coefficients of 0.997. When compared to P-CTX-3C, P-CTX-1 curves expressed a higher toxicity which is shown by having a lower IC_50_ of 0.089 ± 0.011 pg mL^-1^ versus 1.66 ± 0.16 pg mL^-1^ for P-CTX-3C (Table 2). The IC_50_ for P-CTX-1 calculated in this study is similar to previously published values of 0.18 ± 0.11 pg mL^-1^ [21] and 0.078 ± 0.015 pg mL^-1^ [22] (Table 2). Our IC_50_ for P-CTX-3C was higher, yet similar to previous literature values of 0.57 ± 0.11 [21], 1.3 ± 0.06 [17], and 0.91 ± 0.13 pg mL^-1^ [22] (Table 2). Discrepancies can be attributed to different experimental conditions such as various concentrations of ouabain and veratridine used.

**Table 2:** CBA-N2a EC_50_ Values. Literature and this study’s cytotoxicity EC_50_ values of the ciguatoxins P-CTX-3C, P-CTX-1, and C-CTX-1 in association with CBA-N2a. Error is represented as ± standard deviations (SD).

EC_50_ (pg mL^-1^) ± SD			
CBA-N2a	P-CTX-3C	P-CTX-1	C-CTX-1
This study	1.66 ± 0.16	0.089 ± 0.011	
[21]	0.57 ± 0.11	0.18 ± 0.11	0.74 ± 0.11
[17]	1.3 ± 0.06		
[22]	0.91 ± 0.13	0.078 ± 0.015	

**Fig 8:**
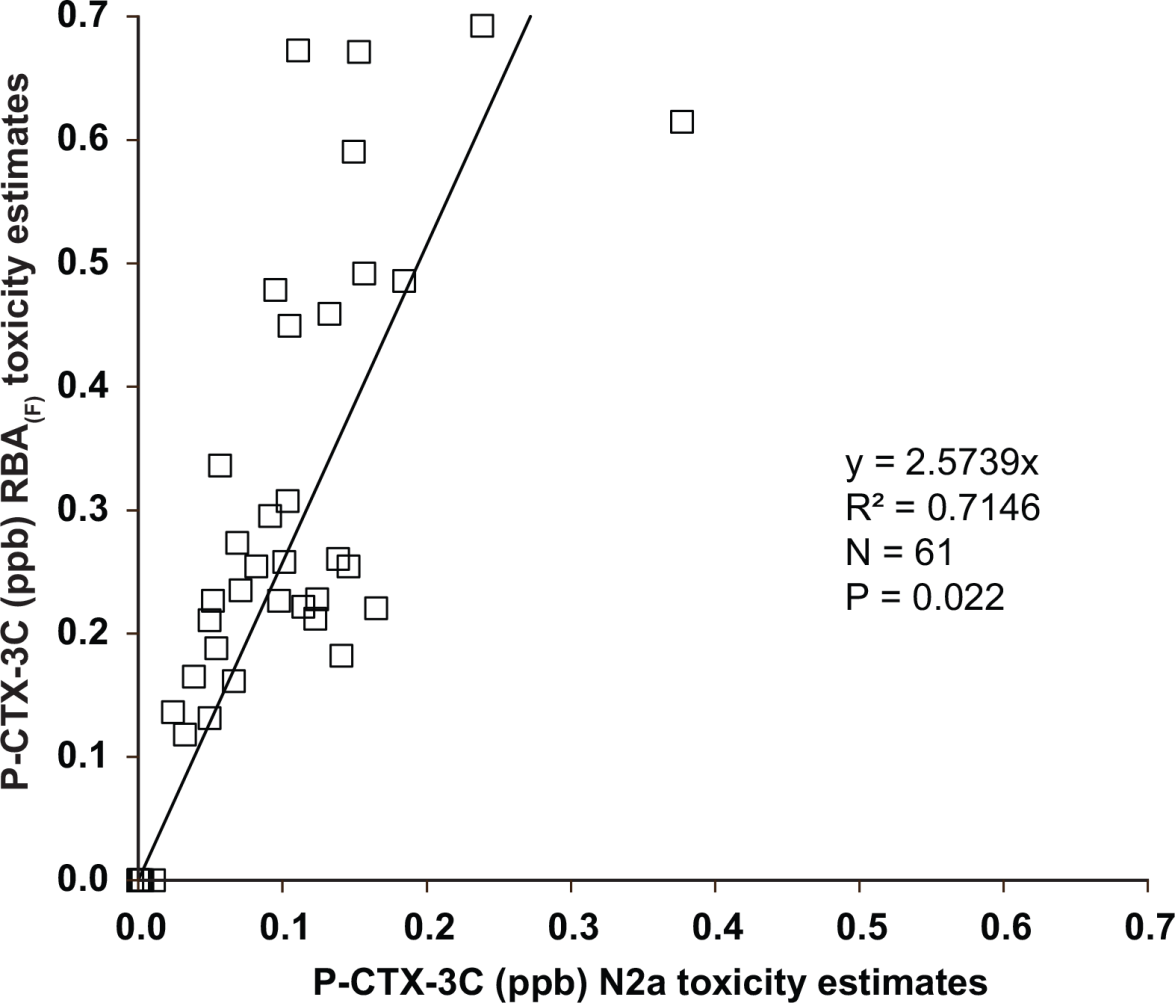
RBA_(F)_ and CBA-N2a Results Comparison. Linear regression between the ciguatoxin concentrations measured in sixty-one duplicate fish extracts using the RBA_(F)_ and CBA-N2a.

All fish extracts that tested positive for CTX-like activity with the RBA_(F)_ (N = 33) were subsequently analyzed by CBA-N2a. In addition, 28 randomly selected fish that were negative by RBA_(F)_ were also tested by CBA-N2a to determine the presence of false negatives. As shown in Fig 8, a t-test proved the regression line of the results between the RBA_(F)_ and CBA-N2a were significant (P = 0.022) with a regression coefficient (R^2^) equal to 0.715. A comparison of the two assays showed that the RBA_(F)_ overestimated the CTX-like CBA-N2a activity of fish extracts by 20–80%. All fish testing positive for CTX by RBA_(F)_ also tested positive by CBA-N2a yet at much lower concentrations (Fig 8). The analysis of negative fish resulted in four false negatives (based upon detection of activity only) of CTX activity by RBA_(F)_ as determined by CBA-N2a. It is noteworthy that these four fish had very low CTX-like activity ranging from 0.002–0.011 ppb which is significantly lower (10–50 fold) than the FDA guidance level 0.1 ppb. These CTX concentrations were near the detection limit of the CBA-N2a, and well below the detection limit (10–50 fold lower) of the RBA_(F)_.

### LC-MS/MS Confirmation

**Fig 9:**
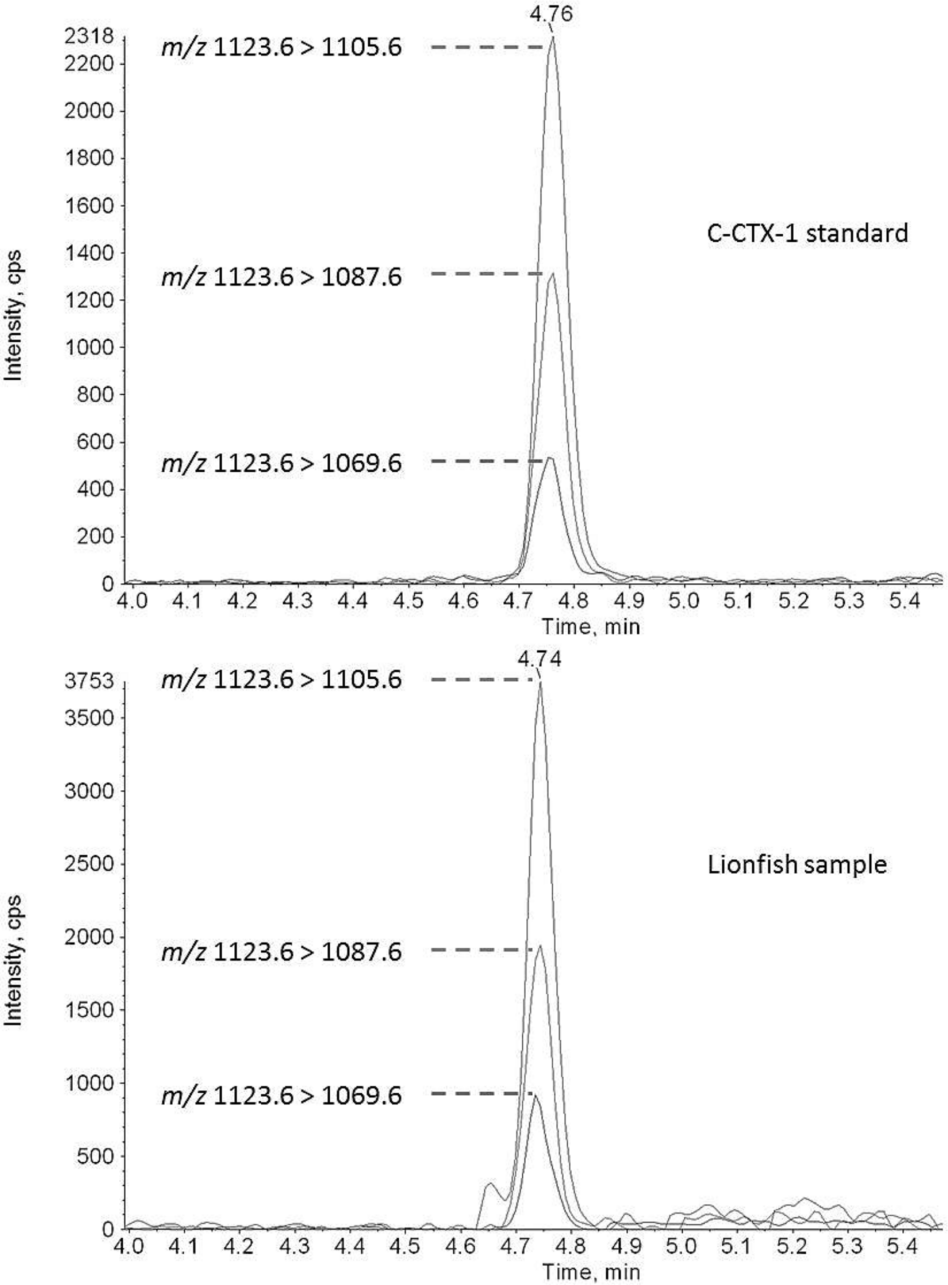
LC-MS/MS Confirmation of Ciguatoxins. LC-MS/MS chromatogram showing the retention time (4.76 min) and the three characteristic ion transitions (1123.6 > 1105.6, 1123.6 > 1087.9, and 1123.6 > 1069.6) of a C-CTX-1 standard and in a lionfish sample.

The presence of C-CTXs in the fish extracts was confirmed by the detection of C-CTX-1 by LC-MS/MS. The retention time and characteristic ion ratios of the C-CTX-1 standard were consistent with those of the sample extracts (Fig 9).

## Discussion

The fluorescent receptor binding assay (RBA_(F)_) developed in this study proved to be a robust screening tool for ciguatoxins in fish. The sensitivity and kinetics of the assay are similar to the established RBA_(R)_ method and provide comparable results without the expense and regulation associated with the use of a radioactive ligand (Table 1; Figs 1–6) [16]. Of the sixty-one fish analyzed by the fluorescent assay, no false positives were observed. All the fish which tested positive by the RBA_(F)_ were found to contain measureable amounts of ciguatoxin activity by the CBA-N2a. The confirmatory analysis of C-CTXs by LC-MS/MS in the fish samples proved that the toxicity determined by RBA_(F)_ and CBA-N2a was not the result of similar compounds such as the proteinaceous scorpaenitoxin that can be found in lionfish [23]. Twenty-eight of the fish that tested negative for CTX activity by the RBA_(F)_ were also analyzed using the CBA-N2a. All but four of these samples contained no measureable CTX as well and were only detected because the CBA-N2a has a much lower detection limit. These remaining four samples contained CTX levels that were considerably below the FDA guidance level of 0.1 ppb C-CTX-1 equivalents. Therefore, relative to the current guidance level, no false negatives were observed. The only disadvantage observed with the RBA_(F)_, and the same is true for the RBA_(R)_, is that the detection limit is higher than the CBA-N2a. However, the fluorescent method is highly applicable to screening of samples relative to the current FDA guidance levels.

Data analysis revealed that the C-CTX-1 equivalents present in the sample measured by the RBA_(F)_ were 20–80% higher than those measured by the CBA-N2a. Essentially, the increased binding affinity of CTX to the receptors translated into a proportionately lower Neuro-2a cytotoxicity. However, there was a significant correlation between the RBA_(F)_ and the CBA-N2a functional assay results (R^2^ = 0.7146; t-test, P = 0.022; Fig 8). Literature comparisons of RBA and CBA-N2a CTX measurements are few. Only one other study reported a similar comparison between the radiolabeled RBA and CBA-N2a [19]. That study showed a similar positive correlation (R^2^ = 0.77) between the two methods with the RBA_(R)_ yielding higher CTX equivalents on average. The variation in the RBA_(F)_: CBA-N2a correlation is likely due to the fact that the fish samples may contain multiple CTX congeners with varying affinities to sodium channels. Consequently, equivalent amounts of bound toxin from the various samples may produce varying degrees of cytotoxicity [24]. Fortunately, the RBA_(F)_ sufficiently overestimates cytotoxicity to such a degree that this variation would not prevent detection of fish containing more than 0.08 ppb C-CTX-1 equivalents by the CBA-N2a (Fig 8). The described overestimation advocates the RBA_(F)_ as an excellent tool for screening samples fast and efficiently.

In this study, the rapid screening method, using the 0 to 1.0 ppb P-CTX-3C equivalents standard curve, was able to quantify the C-CTX-1 equivalents in every fish, except the one which contained > 1.0 ppb. The linear standard curve approach is advantageous in that it allows screening of up to 18 samples on a single plate and eliminates the need for serial sample dilutions. This approach increases laboratory efficiency and reduces per sample screening costs. Samples containing higher CTX concentrations (> 1 ppb) can be accurately screened by preparing a sample dilution curve, estimating the IC_50_, and converting that IC_50_ into a toxicity estimate.

In summary, the RBA_(F)_ can be used to rapidly screen fish for the presence of ciguatoxins. The assay takes < 3 h to complete, is stable over long periods of time, requires relatively little purified toxin standards, generates no radioactive waste, and is cost effective. Also, current extraction protocols being used for the RBA_(R)_ can be similarly used for the RBA_(F)_ with only the addition of a simple cooking step. The RBA_(F)_ exhibits similar binding kinetics as the RBA_(R)_ already in use and correlates well with the CBA-N2a. Most fluorescent plate readers, which are relatively common laboratory instruments, can be adapted to perform the RBA_(F)_ assay, reducing startup costs. The assay is ideal for incorporation into routine CTX monitoring programs and is currently being employed in our laboratory to estimate CTX levels in Caribbean lionfish to determine whether implementation of a fishery can be done in reef communities without public health concerns. Even though this study focused on Caribbean samples, the RBA_(F)_ is highly applicable to Pacific ciguatoxins in fish as well as phytoplankton samples and will be the focus of future studies in widespread implementation of this assay worldwide.
